# Omega-3 fatty acids and oxidative stability of ice cream supplemented with olein fraction of chia (*Salvia hispanica* L.) oil

**DOI:** 10.1186/s12944-017-0420-y

**Published:** 2017-02-07

**Authors:** Rahman Ullah, Muhammad Nadeem, Muhammad Imran

**Affiliations:** 1 Department of Dairy Technology, University of Veterinary and Animal Sciences, Lahore, Pakistan; 2 Institute of Home and Food Sciences, Faculty of Science and Technology, Government College University, Faisalabad, Pakistan

**Keywords:** Omega-3 fatty acids, Olein fraction, Phenolic contents, Flavonoids, Oxidative stability

## Abstract

**Background:**

Chia (*Salvia hispanica* L.) has been regarded as good source of polyunsaturated omega-3 fatty acids with cardiac, hepatic, hypotensive, antiallergic and antidiabetic role. Concentration of omega-3 fatty acids in chia oil can be enhanced by fractionation. Olein/low melting fraction of chia oil has higher concentration of omega-3 fatty acids. Therefore, main objective of current investigation was determination of various concentration effect of olein fraction of chia oil on omega-3 fatty acids, oxidative stability and sensory characteristics of ice cream.

**Methods:**

Ice cream samples were prepared by partially replacing the milk fat with olein fraction of chia oil at 5, 10, 15 and 20% concentrations (T_1_, T_2_, T_3_ and T_4_), respectively. Ice cream prepared from 100% milk fat was kept as control. Ice cream samples stored at −18 °C for 60 days were analysed at 0, 30 and 60 days of the storage period. Fatty acid profile, total phenolic contents, total flavonoids, free fatty acids, peroxide value, anisidine value and sensory characteristics of ice cream samples was studied.

**Results:**

Concentration of α-linolenic acid, eicosapentaenoic acid, docosapentaenoic acid and docosahexaenoic acid in T_4_ was 13.24, 0.58, 0.42 and 0.31%, respectively. Total phenolic contents of control, T_1_, T_2_, T_3_ and T_4_ were recorded 0.12, 1.65, 3.17, 5.19 and 7.48 mg GAE/mL, respectively. Total flavonoid content of control, T_1_, T_2_, T_3_ and T_4_ were found 0.08, 0.64, 1.87, 3.16 and 4.29 mg Quercetin Equivalent/mL. 2,2-diphenyl-1-picrylhydrazyl (DPPH) free radical scavenging activity of control, T_1_, T_2_, T_3_ and T_4_ was noted 5.61, 17.43, 36.84, 51.17 and 74.91%, respectively. After 60 days of storage period, the highest peroxide value of 1.84 (MeqO_2_/kg) was observed in T_4_, which was much less than allowable limit of 10 (MeqO_2_/kg). Flavour score was non-significant after 30 days of storage period.

**Conclusions:**

Supplementation of ice cream with olein fraction of chia oil enhanced the concentration of omega-3 fatty acids and improved the antioxidant perspectives of ice cream. These results suggest that omega-3 fatty acids and antioxidant characteristics of ice cream may be improved with olein fraction of chia oil for discerning consumers.

## Background

Cardiovascular disease is one of most leading eradicator of mankind and according to the British Heart Network, the rise in deaths from cardiovascular diseases may reach up to 25 million/year in 2025. Scientific evidences have revealed that polyunsaturated fatty acids have beneficial impact on cardiovascular system [1]. Omega-3 fatty acids are polyunsaturated fatty acids and Directions of American Heart Association also recommends the consumers to increase the intake of omega-3 fatty acids [2]. Cardio, hepatic, hypotensive, antiallergic and antidiabetic importance of omega-3 fatty acids are scientifically proven [3]. In infants, omega-3 fatty acids play a vital role in the development of brain, cardiovascular system and eyes [4]. The knowledge of nutrition and health related sicknesses have great deal of impact on the consumption patterns. Consumers have become more health conscious and growing interest of consumers towards the functional foods has led the food industry to increase the functional value of dairy products [5]. Fish is regarded as superb source of omega-3 fatty acids, however, fish oil and fish powder may carry undesirable fishy flavour to the food products [6]. The enhancement of ice cream nutritional value through fish powder has not been recommended for industrial applications due to the persistence of fishy flavour of oil and powder. In such a situation, potential source of omega-3 fatty acids should be discovered for easy adaptation and better industrial application. Chia (*Salvia hispanica* L.) produces about 35–40% superior quality edible oil, which contains about 63–65% omega-3 fatty acids [7]. Chia seeds have been the part of human food since 1500 BC. Chia has been declared as Novel Food with no anti-nutritional factors [8]. The concentration of omega-3 fatty acids in olein fraction of chia oil was more than 80%, which is highest in all the known foods [9]. Oxidative stability of food products is also an extremely important consideration while enhancing the nutritional value of foods. Ice cream is a popular product among the people of all ages around the world. Numerous studies have been performed to enhance the concentration of unsaturated fatty acids in ice cream. Oxidative stability of ice cream with higher concentration of unsaturated fatty acids was less than standard ice cream [10]. Modified versions of other dairy products have lower oxidative stability [11]. The concentration of unsaturated fatty acids in ice cream prepared from the milk of cows fed on calcium salts of fatty acids was greater than standard ice cream [12]. Concentration of unsaturated fatty acids in ice cream prepared from low melting fractions of milk fat has also been found higher than ice cream prepared from unmodified milk fat [13]. Addition of flaxseed oil increased the unsaturated fatty acids in ice cream; however, oxidative stability of ice cream was lesser than control [14]. Studies on dry and solvent fractionation have shown that dry fractionation is superior to solvent fractionation for better industrial application and safety perspectives [15]. Oxidative stresses may lead to diabetes, development of cancer, DNA mutation, atherogenesis and accelerated ageing. For the prevention of oxidative stresses in body, antioxidant should be present in sufficient concentration. Numerous studies have been conducted to increase the concentration of unsaturated fatty acids in ice cream by replacing milk fat with vegetable oils and fats while little is known regarding the supplementation of ice with omega-3 fatty acids through olein fraction of chia oil. This study was aimed to enhance the concentration of omega-3 fatty acids and oxidative stability of ice cream on the basis of chemical and sensory characteristics.

## Methods

### Materials

Skim milk powder, sugar, butter, cremodan and chia seeds were purchased from local market. All the chemicals used in this investigation were HPLC grade and procured from Sigma Aldrich, St. Louis, Mo, USA.

### Chemical characteristics of chia seed and oil

Proximate composition of chia seed was determined by following the standard methods [16]. Chia oil was characterized for free fatty acids, moisture content, iodine value, saponification value, unsaponifiable matter [17]. Colour was determined on a Lovibond Tintometer (Tintometer Corporation, Salisbury, England).

### Oil extraction and preparation of olein fraction of chia oil

Oil from chia seeds was extracted by a laboratory scale expeller. For the preparation of olein fraction, chia oil was heated to 63 °C, gradually cooled to −30 °C in 2 h, held for further 5 h, followed by pressure filtration using Buckner Flask at −600 mmHg pressure. Filtrate was regarded as olein fraction. Processes of fractionation were repeated 6 times, filtrates were pooled, stored in amber glass bottle at −30 °C, till further usage in current investigation [15].

### Experimental plan

Experiment was planned in a completely randomized design (CRD); each treatment was run three times. Milk fat was partially replaced with olein fraction of chia oil at 5, 10, 15 and 20% concentrations (T_1_, T_2_, T_3_ and T_4_), respectively. Ice cream prepared from 100% milk fat served as control. All types of ice creams contained 11% SNF, 10%, milk fat, 13% sugar and 0.5% cremodan. Ice cream mix was pasteurized at 85 °C, for 1 min, aged at 4 °C, for 16 h, whipped and stored at −18 °C for 60 days. Chemical and sensory characteristics were determined at 0, 30 and 60 days of the storage period.

### Fatty acid profile

Fatty acid profile was determined by transforming the fat to fatty acid methyl esters, which were prepared by reacting 50 mg fat with 2 mL (15% methanolic HCl, Fluka) at 100 °C, for 1 h, tubes were cooled to room temperature, 2 mL *n*-hexane and 2 mL deionized water were added, vortexed at 500 × g for 2 min. Test tubes were allowed to stand for 15 min; supernatant was transferred to GC vials, injected to GC-MS (7890 A GC System Agilent) fitted MSD detector, using ZB-5 fused silica capillary column (Zebron Phenomenex, 30 m × 0.25 mm) [18]. Fatty acids were identified and quantified by FAME 37 Kit, Sigma-Aldrich, Chemical Company.

### Antioxidant characteristics of ice cream

Evaluation of antioxidant characteristics of ice samples were done with the aid of following tests.

### Total phenolic contents

Total phenolic contents of ice creams were determined by following the method [19]. Ice cream (0.1 mg) was mixed with 5.9 mL deionized water, 1 mL of the diluted ice cream sample was mixed with 1 mL Folin-Ciocateu reagent, after 5 min of standing, 2 mL of 20% (w/v) sodium carbonate was added. Contents of test tube were stirred and incubated at room temperature for 10 min, followed by homogenization at 1500 × g. Absorbance was measured on a double beam spectrophotometer (Shimadzu, Japan) at 550 nm. Total phenolic contents were determined from the calibration curve using Gallic acid as standard (10–100 ppm) and reported as GAE (mg/g). Value of R^2^ for each determination was not less than 0.9872.

### Total flavonoid contents

Total flavonoid content of ice cream was determined according to the colorimetric method using Rutin as standard [20]. AlCl_3_ (2% solution) was prepared in methanol, then 0.5 mL sample was mixed with 0.5 mL AlCl_3_, followed by incubation at room temperature for 1 h, absorbance was measured at 420 nm. Total flavonoid contents were calculated from the following formula and reported as quercetin equivalent (mg/g).

Total flavonoid contents (Quercetin Equivalent mg/g) = 0.025 × Absorbance.

### DPPH free radical scavenging activity

The DPPH free radical scavenging activity of ice cream supplemented with olein fraction of chia oil was determined according to the method [21]. DPPH solution (0.1 mM) was prepared in methanol, 2.9 mL of DPPH solution was mixed to sample (100 μL), vortexed in screw capped test tube at 500 × g for 2 min, tubes were incubated at room in dark for 30 min. Absorbance was measured on a double beam spectrophotometer (Shimadzu, Japan) at 517 nm in visible region of spectrum.

### Oxidative stability and sensory evaluation

Free fatty acids (oleic), peroxide and anisidine values were determined according to the standard methods [17]. Sensory evaluation of ice cream supplemented with olein fraction of chia oil was performed by following the standard method of the American Oil Chemists Society [22].

### Statistical analysis

One way and two way analysis of variance techniques were used to find out the effect of treatment, storage and their interaction. Duncan Multiple Range Test was used to denote the significant difference among the treatments on SAS 9.1 statistical software. Results were declared significant al *p*-value 0.05 (P ≤ 0.05) [23].

## Results

The supplementation of ice cream with olein fraction of chia oil did not have any effect on fat, protein, total solids and pH of ice cream (Table 1) (*P* > 0.05). Results of fatty acid profile of ice cream supplemented with olein fraction of chia oil are presented in Table 2. Blending of milk fat with olein fraction of chia oil altered the omega-3 fatty acids composition of milk fat. α-linolenic acid (ALA) was present 0.31% in milk fat, whereas, eicosapentaenoic acid (EPA), docosapentaenoic acid (DPA) and docosahexaenoic acid (DHA) was not detected in milk fat. Concentration of ALA, EPA, DPA and DHA in T_3_ was 10.19, 0.41, 0.29 and 0.23%, whereas in T_4_ was 13.24, 0.58, 0.42 and 0.31%, respectively. In T_4_, after 60 days of storage period, concentration of ALA, EPA, DPA and DHA decreased by 0.78, 0.08, 0.02 and 0.14%, respectively (Table 3). Total phenolic contents of control, T_1_, T_2_, T_3_ and T_4_ samples were recorded 0.12, 1.65, 3.17, 5.19 and 7.48 mg GAE/mL, respectively while total phenolic contents of olein fraction of chia oil were observed 13.61 mg GAE/mL. Total flavonoid content of control, T_1_, T_2_, T_3_ and T_4_ were noted as 0.08, 0.64, 1.87, 3.16 and 4.29 mg Quercetin Equivalent/mL, respectively. IC_50_ values T_1_, T_2_, T_3_ and T_4_ for the inhibition of lipid peroxidation were 52 μg/mL, 3852 μg/mL, 2552 μg/mL and 1152 μg/mL, respectively. DPPH free radical scavenging activity of control, T_1_, T_2_, T_3_ and T_4_ was found 5.61, 17.43, 36.84, 51.17 and 74.91%, respectively.

**Table 1:** Chemical Composition of Ice Cream Supplemented with Olein Fraction of Chia Oil

Treatments	Fat %	Protein %	Total solids %	pH	Overrun %
Control	9.81 ± 0.24^a^	4.11 ± 0.05^a^	34.2 ± 0.26^a^	6.67 ± 0.15^a^	85.7 ± 2.65^a^
T_1_	9.63 ± 0.19^a^	4.15 ± 0.09^a^	34.4 ± 0.41^a^	6.65 ± 0.09^a^	84.2 ± 3.12^a^
T_2_	9.91 ± 0.15^a^	3.97 ± 0.15^a^	34.7 ± 0.34^a^	6.71 ± 0.13^a^	82.5 ± 1.58^a^
T_3_	9.48 ± 0.13^a^	4.05 ± 0.11^a^	34.8 ± 0.61^a^	6.62 ± 0.16^a^	78.3 ± 4.53^b^
T_4_	9.73 ± 0.28^a^	4.09 ± 0.07^a^	34.5 ± 0.18^a^	6.63 ± 0.11^a^	75.6 ± 2.87^c^

Within a column, means denoted by a similar letter statistically non-significant (*p* > 0.05)

Control: 100% Milk Fat

T_1_: 95% Milk Fat and 5% Chia Oil

T_2_: 90% Milk Fat and 10% Chia Oil

T_3_: 85% Milk Fat and 15% Chia Oil

T_4_: 80% Milk Fat and 20% Chia Oil

**Table 2:** Fatty Acid Profile of Ice Cream Supplemented with Olein Fraction of Chia Oil

Fatty acid	Control	T_1_	T_2_	T_3_	T_4_
C_4:0_	1.75 ± 0.05^a^	1.64 ± 0.08^b^	1.58 ± 0.04^c^	1.52 ± 0.07^d^	1.45 ± 0.11^e^
C_6:0_	2.18 ± 0.04^a^	2.08 ± 0.12^b^	1.95 ± 0.08^c^	1.84 ± 0.05^d^	1.77 ± 0.03^e^
C_8:0_	2.44 ± 0.07^a^	2.32 ± 0.03^b^	2.15 ± 0.06^c^	2.04 ± 0.01^d^	1.91 ± 0.05^e^
C_10:0_	2.37 ± 0.14^a^	2.26 ± 0.07^b^	2.14 ± 0.09^c^	1.98 ± 0.12^d^	1.84 ± 0.02^e^
C_12:0_	2.76 ± 0.19^a^	2.61 ± 0.11^b^	2.48 ± 0.19^c^	2.31 ± 0.15^d^	2.14 ± 0.14^e^
C_14:0_	10.22 ± 0.34^a^	9.71 ± 0.45^b^	9.19 ± 0.51^c^	8.68 ± 0.26^d^	8.17 ± 0.19^e^
C_16:0_	31.56 ± 0.88^a^	30.32 ± 0.75^a^	29.08 ± 0.31^b^	27.84 ± 0.67^c^	26.41 ± 0.37^d^
C_18:0_	10.35 ± 0.42^a^	9.94 ± 0.13^a^	9.56 ± 0.25^a^	9.21 ± 0.21^a^	8.83 ± 0.33^b^
C_18:1_	23.57 ± 0.73^a^	22.31 ± 0.73^b^	21.16 ± 0.82^c^	19.92 ± 0.53^d^	18.85 ± 0.25^e^
C_18:2_	2.61 ± 0.08^a^	2.47 ± 0.07^b^	2.31 ± 0.12^c^	2.21 ± 0.16^d^	2.05 ± 0.08^e^
α-Linolenic acid	0.31 ± 0.04^e^	3.54 ± 0.10^d^	6.77 ± 0.09^c^	10.19 ± 0.23^b^	13.24 ± 0.28^a^
Eicosapentaenoic acid	ND	0.12 ± 0.02^d^	0.26 ± 0.06^c^	0.41 ± 0.03^b^	0.58 ± 0.04^a^
Docosapentaenoic acid	ND	0.08 ± 0.01^d^	0.18 ± 0.03^c^	0.29 ± 0.05^b^	0.42 ± 0.02^a^
Docosahexaenoic acid	ND	0.11 ± 0.02^d^	0.16 ± 0.01^c^	0.23 ± 0.02^b^	0.31 ± 0.04^a^

*Abbreviations*: *ND* not detected

Means of triplicate experiments and triplicate analysis, within a row means expressed by a different letter are statistically significant (*P* < 0.05)

Control: 100% Milk Fat

T_1_: 95% Milk Fat and 5% Chia Oil

T_2_: 90% Milk Fat and 10% Chia Oil

T_3_: 85% Milk Fat and 15% Chia Oil

T_4_: 80% Milk Fat and 20% Chia Oil

**Table 3:** Transition in Fatty Acid Profile of Ice Cream Supplemented with Olein Fraction of Chia Oil

Fatty acid	Control		T_1_		T_2_		T_3_		T_4_	
	Fresh	60-Days*	Fresh	60-Days*	Fresh	60-Days*	Fresh	60-Days*	Fresh	60-Days*
C_4:0_	1.75 ± 0.05^a^	1.71 ± 0.03^a^	1.64 ± 0.08^b^	1.62 ± 0.05^b^	1.58 ± 0.04^b^	1.49 ± 0.03^c^	1.52 ± 0.07^b^	1.45 ± 0.11^c^	1.44 ± 0.11^c^	1.37 ± 0.06^d^
C_6:0_	2.18 ± 0.04^a^	2.1350.02^a^	2.08 ± 0.12^b^	2.04 ± 0.08^b^	1.95 ± 0.08^c^	1.88 ± 0.04^d^	1.84 ± 0.05^d^	1.77 ± 0.08^e^	1.75 ± 0.03^e^	1.62 ± 0.09^f^
C_8:0_	2.44 ± 0.07^a^	2.38 ± 0.06^a^	2.32 ± 0.03^b^	2.26 ± 0.03^b^	2.15 ± 0.06^c^	2.11 ± 0.18^c^	2.04 ± 0.01^d^	1.96 ± 0.13^e^	1.91 ± 0.05f	1.78 ± 0.05^g^
C_10:0_	2.37 ± 0.14^a^	2.24 ± 0.08^b^	2.26 ± 0.07^b^	2.19 ± 0.14^c^	2.14 ± 0.09^c^	2.09 ± 0.03^d^	1.98 ± 0.12^e^	1.89 ± 0.17^f^	1.84 ± 0.02^f^	1.75 ± 0.03^g^
C_12:0_	2.76 ± 0.19^a^	2.65 ± 0.12^b^	2.61 ± 0.11^b^	2.55 ± 0.28^c^	2.48 ± 0.19^d^	2.40 ± 0.21^e^	2.31 ± 0.15f	1.22 ± 0.03^g^	2.14 ± 0.14^h^	2.11 ± 0.02^h^
C_14:0_	10.22 ± 0.34^a^	9.84 ± 0.15^b^	9.71 ± 0.45^b^	9.61 ± 0.74^b^	9.19 ± 0.51^c^	8.94 ± 0.78d	8.68 ± 0.26^e^	8.54 ± 0.26^e^	8.17 ± 0.19^f^	7.98 ± 0.16^g^
C_16:0_	31.56 ± 0.88^a^	30.14 ± 0.98^b^	30.32 ± 0.75^b^	29.81 ± 1.27^c^	29.08 ± 0.31^c^	28.11 ± 1.36^d^	27.84 ± 0.67^e^	26.19 ± 1.16^f^	26.41 ± 0.88^f^	25.04 ± 1.37^g^
C_18:0_	10.35 ± 0.42^a^	10.26 ± 0.24^a^	9.94 ± 0.13^b^	9.76 ± 0.57^b^	9.56 ± 0.25^c^	9.34 ± 0.43^c^	9.21 ± 0.21^d^	8.93 ± 0.35^e^	8.83 ± 0.33^e^	8.61 ± 0.53^f^
C_18:1_	23.57 ± 0.73^a^	21.66 ± 0.82^c^	22.31 ± 0.73^b^	20.48 ± 0.94^c^	21.16 ± 0.82^c^	19.67 ± 0.81^d^	19.92 ± 0.61^d^	18.27 ± 0.42^e^	18.85 ± 0.25^e^	16.39 ± 0.39^f^
C_18:2_	2.71 ± 0.08^a^	1.39 ± 0.13^g^	2.47 ± 0.07^b^	1.27 ± 0.03^h^	2.31 ± 0.12^c^	1.41 ± 0.05^g^	2.21 ± 0.16^d^	1.75 ± 0.12^f^	2.05 ± 0.08^e^	1.35 ± 0.08^g^
α-Linolenic	ND	ND	3.54 ± 0.02^g^	3.19 ± 0.02^h^	6.77 ± 0.06^e^	6.20 ± 0.02^f^	10.19 ± 0.03^c^	9.35 ± 0.06^d^	13.24 ± 0.04^a^	12.46 ± 0.04^b^
Eicosapentaenoic acid	ND	ND	0.08 ± 0.01^g^	0.06 ± 0.01^g^	0.18 ± 0.03^e^	0.14 ± 0.06^f^	0.29 ± 0.05^c^	0.24 ± 0.03^d^	0.42 ± 0.02^a^	0.34 ± 0.02^b^
Docosapentaenoic acid	ND	ND	0.11 ± 0.02^d^	0.07 ± 0.01^e^	0.16 ± 0.01^c^	0.11 ± 0.02^d^	0.23 ± 0.02^b^	0.18 ± 0.02^c^	0.31 ± 0.04^a^	0.29 ± 0.01^a^
Docosahexaenoic acid	ND	ND	1.64 ± 0.08^a^	1.56 ± 0.22^b^	1.58 ± 0.04^b^	1.42 ± 0.03^c^	1.52 ± 0.07^b^	1.39 ± 0.01^c^	1.45 ± 0.11^c^	1.31 ± 0.09^d^

*Abbreviations*: *ND* not detected

*60 Days Stored; Means of triplicate experiments; within a row means denoted with a different letter are statistically different (*p* < 0.05)

Control: 100% Milk Fat

T_1_: 95% Milk Fat and 5% Chia Oil

T_2_: 90% Milk Fat and 10% Chia Oil

T_3_: 85% Milk Fat and 15% Chia Oil

T_4_: 80% Milk Fat and 20% Chia Oil

Results of oxidative stability of ice cream added with olein fraction of chia oil are given in Table 4. In current investigation, free fatty acids of olein fraction and butter fat were 0.14 and 0.11%, respectively. Lower free fatty acids in substrate oil and fat led to the lower content of free fatty acids in ice creams. Free fatty acids of all the treatments and control ranged from 0.08 to 0.12%. Free fatty acids of all the treatments and control slowly increased during the storage period of 60 days. After 60 days of storage period, free fatty acids of control and T_4_ were 0.14 and 0.16%, respectively (*P* > 0.05). Free fatty acids of ice cream supplemented with various levels of olein fraction of chia oil were less than allowable limit of 0.2%. Anisidine value determines the secondary and tertiary stages of auto-oxidation. During the storage period, rise in peroxide and anisidine values was recorded. Storage period with respect to peroxide value and anisidine value was found non-significant for all the treatments and control. After 60 days of storage period, T_3_ and T_4_ underwent more oxidation as compared to the control and other treatments. After 60 days of storage period, highest peroxide value of 1.84 (MeqO_2_/kg) was observed in T_4_ which is much less than allowable limit of 10 (MeqO_2_/kg).

**Table 4:** Oxidative Stability of Ice Cream Supplemented with Olein Fraction of Chia Oil

Treatments	Storage days	FFA %	PV (MeqO_2_/kg)	AV
Control	0 30 60	0.08 ± 0.01^a^ 0.11 ± 0.02^a^ 0.14 ± 0.04^b^	0.26 ± 0.03^f^ 0.33 ± 0.05^f^ 0.61 ± 0.02^e^	4.72 ± 0.19^f^ 4.82 ± 0.24^f^ 7.39 ± 0.31^e^
T_1_	0 30 60	0.10 ± 0.02^a^ 0.12 ± 0.03^a^ 0.15 ± 0.01^b^	0.25 ± 0.04^f^ 0.27 ± 0.07^f^ 0.74 ± 0.09^d^	4.75 ± 0.08^f^ 4.61 ± 0.21^f^ 7.56 ± 0.29^d^
T_2_	0 30 60	0.11 ± 0.01^a^ 0.13 ± 0.03^a^ 0.16 ± 0.01^b^	0.25 ± 0.03^f^ 0.29 ± 0.02^f^ 0.92 ± 0.08^c^	4.78 ± 0.04^f^ 4.69 ± 0.17^f^ 10.63 ± 0.42^c^
T_3_	0 30 60	0.11 ± 0.02^a^ 0.12 ± 0.04^a^ 0.16 ± 0.01^b^	0.27 ± 0.09^f^ 0.32 ± 0.06^f^ 1.28 ± 0.11^b^	4.82 ± 0.27^f^ 4.80 ± 0.32^f^ 13.44 ± 0.44^b^
T_4_	0 30 60	0.12 ± 0.01^a^ 0.13 ± 0.02^a^ 0.16 ± 0.05^b^	0.31 ± 0.02^f^ 0.35 ± 0.05^f^ 1.84 ± 0.14^a^	4.88 ± 0.14^f^ 4.68 ± 0.51^f^ 17.69 ± 0.91^a^

*Abbreviations*: *FFA* free fatty acids (Oleic Acid), *PV* peroxide value, *AV* anisidine value

Within a column, means denoted by a common letter are statistically non-significant (*P* > 0.05)

Control: 100% Milk Fat

T_1_: 95% Milk Fat and 5% Chia Oil

T_2_: 90% Milk Fat and 10% Chia Oil

T_3_: 85% Milk Fat and 15% Chia Oil

T_4_: 80% Milk Fat and 20% Chia Oil

Results of sensory evaluation of ice cream supplemented with olein fraction have been presented in Table 5. Addition of olein fraction of chia oil did not have any impact on colour, flavour and texture of fresh ice cream. Colour, flavour and texture score was non-significant up to 30 days of storage period. After 30 days, sensory score deteriorated and decline in sensory score was not due to the addition of olein fraction of chia oil rather it was due to the oxidation of unsaturated fatty acids. Peroxide value and taste score were strongly correlated (*R* ^*2*^ = 0.998). Decline in flavour score of ice cream prepared from olein fraction of milk fat was due to the generation of peroxides during the storage period [13].

**Table 5:** Sensory Characteristics of Ice Cream Supplemented with Olein Fraction of Chia Oil

Treatments	Storage days	Colour	Flavour	Texture
Control	0 30 60	8.2 ± 0.18^a^ 8.0 ± 0.14^a^ 7.6 ± 0.29^b^	8.0 ± 0.23^a^ 7.9 ± 0.21^a^ 7.6 ± 0.25^b^	8.2 ± 0.19^a^ 8.0 ± 0.25^a^ 7.9 ± 0.21^a^
T_1_	0 30 60	8.1 ± 0.16^a^ 7.8 ± 0.15^a^ 7.5 ± 0.24^b^	8.2 ± 0.24^a^ 8.0 ± 0.19^a^ 7.5 ± 0.20^b^	8.1 ± 0.20^a^ 7.9 ± 0.23^a^ 7.8 ± 0.19^a^
T_2_	0 30 60	8.0 ± 0.14^a^ 7.9 ± 0.17^a^ 7.4 ± 0.23^b^	8.1 ± 0.15^a^ 7.8 ± 0.14^a^ 7.0 ± 0.22^b^	8.1 ± 0.20^a^ 7.6 ± 0.10^b^ 7.3 ± 0.14^c^
T_3_	0 30 60	8.1 ± 0.20^a^ 7.8 ± 0.14^a^ 7.2 ± 0.21^b^	8.1 ± 0.23^a^ 7.9 ± 0.13^a^ 6.9 ± 0.18^b^	7.9 ± 0.20^a^ 7.7 ± 0.16^b^ 7.1 ± 0.19^d^
T_4_	0 30 60	7.9 ± 0.18^a^ 7.6 ± 0.13^a^ 7.1 ± 0.22^b^	7.8 ± 0.30^a^ 7.9 ± 0.21^a^ 6.5 ± 0.24^c^	8.0 ± 0.20^a^ 7.8 ± 0.18^a^ 7.0 ± 0.20^d^

Within a column, means denoted by a common letter are statistically non-significant (*P* > 0.05)

Control: 100% Milk Fat

T_1_: 95% Milk Fat and 5% Chia Oil

T_2_: 90% Milk Fat and 10% Chia Oil

T_3_: 85% Milk Fat and 15% Chia Oil

T_4_: 80% Milk Fat and 20% Chia Oil

## Discussion

### Effect of olein fraction on fatty acid profile

Non-significant impact on compositional attributes of ice cream may be connected to the identical ingredients and processing conditions for all the treatments. Earlier studies also suggested non-significant effect of unsaturated fatty acids on compositional attributes of ice cream [12]. Overrun of ice cream supplemented with 15 and 20% olein fraction of chia oil were less than control. Overrun of ice cream mainly depends upon fatty acid composition of fat used in ice cream [10]. Addition of unsaturated fatty acids in ice cream resulted in lower overrun of ice cream [13]. Earlier investigations have shown that blending of milk fat with vegetable oils had pronounced effect fatty acid profile of fats and oils [24, 25]. Partial replacement of milk fat with flaxseed oil enhanced the concentration of beneficial unsaturated fatty acids [15]. Role of omega-3 fatty acids as cardiac, hepatic protective, anti-inflammatory, brain and eye developers have been scientifically established. With greater than 72% α-linolenic acid, olein fraction of chia oil may be regarded as the richest source of α-linolenic acid [9]. Olein fraction of chia oil may open new avenues for increasing functional value of foods. It may be used as superb source of omega-3 fatty acids in several food items. Furthermore, the ratio of omega-3 and 6 fatty acids can be balanced in the diet by supplementing with olein fraction of omega-3 enriched oil. However, this requires detailed further investigation.

### Changes in fatty acid profile during storage

During the enhancement of nutritional value, it is extremely important take into consideration the oxidative stability of fat based foods. Foods having higher concentration of unsaturated fatty acids are susceptible to auto-oxidation. In current investigation, storage effect on fatty acid profile of ice cream was used as an indication of oxidative stability. Earlier studies have also evidenced that monitoring of the fatty acid profile of ice cream during the storage period provided better indication of the oxidative stability [13, 26]. Storage stability of dairy products with modified fatty acid profile was less than standard products [27]. The existence of chlorogenic acid, caffeic acid, quercetin, phenolic glycoside-k and phenolic glycoside-Q phenolic compounds in olein fraction of chia oil efficiently inhibited the breakdown of fatty acids into food products [9, 26]. Oxidative stability of dairy products with modified fatty acid profile was different from standard products [27–29].

### Effect of olein fraction on total phenolic and flavonoids contents

Numerous disorders such as, atherosclerosis, arthritis and cancer in organs of human body may be due to higher concentration of free radicals [30]. Scientific evidences have shown that secondary metabolites of plant have pharmacological and biological activity against oxidative stress, cancer, accelerated ageing and atherogenesis [31, 32]. This could be the probable reason for higher phenolic contents in experimental samples. Total phenolic contents of olein fraction of chia oil were greater than commonly used vegetable oils [33]. Flavonoids are extremely active scavengers of large number of reactive oxygen species. Antioxidant characteristics of ice cream have been enhanced by sugarcane juice, however, little is known regarding the boosting of antioxidant characteristics of ice cream through vegetable oil [34].

### Effect of olein fraction on DPPH free radical scavenging activity

Lipid oxidation has been renowned as a most important problem in the storage of dairy products having unsaturated fatty acids. Oxidative deterioration can consequence in off flavours, obliteration of nutrients, bioactive compounds and generation of potentially toxic oxidation products [35]. DPPH free radical scavenging activity is recognized as one of the most parameter to assess the antioxidant characteristics of natural antioxidants. Addition of *Kitaibelia vitifolia* extract in Pirotski Kachkaval cheese enhanced the DPPH free radical scavenging activity [36]. El-Din et al. [37] studied the effect of rosemary extract on antioxidant characteristics of ice cream on the basis of total phenolic contents and DPPH free radical scavenging activity. Addition of rosemary extract increased the total phenolic contents, DPPH free radical scavenging activity and shelf life of cheese. Supplementation of cheese with rosemary extract improved the antioxidant characteristics [38].

### Effect of olein fraction on oxidative stability

Free fatty acids are generated as a result of hydrolysis of triglycerides and moisture, lipases, storage temperature and metal ions are regarded as accelerators of hydrolysis [39]. Free fatty acids affect the quality characteristics of fat based foods in two ways such as they induce objectionable flavours and secondly they accelerate the breakdown of peroxides to oxidation products. Manufacturers of edible oils regard free fatty acids as undesirable compounds for processing and subsequent storage. Oils with lower free fatty acids obtain higher market prices with better industrial processing and higher storage stability [40]. Classical rise in free fatty acids of butter during storage has also been recorded [41]. Free fatty acids of olein fraction of *Moringa oleifera* oil increased during the during storage period [42]. Estimation of peroxide value gives useful information regarding the oxidation status of oils and fats. Food stuffs with lower peroxide value usually have better shelf life with stable flavour profile. Peroxide value of ice cream increased during the storage period [10]. Lipid oxidation in ice cream was efficiently inhibited by phenolic compounds of chia oil. Our earlier investigation regarding the HPLC characterization of phenolic compounds of olein fraction of chia oil, chlorogenic acid, caffeic acid, quercetin, phenolic glycoside-k and phenolic glycoside-Q were identified in reasonable amounts. Antioxidant activity of these phenolic compounds is well established [43].

## Conclusions

Supplementation of ice cream with olein fraction of chia oil significantly improved the concentration of omega-3 fatty acids in ice cream. Total phenolic contents, total flavonoids and DPPH free radical scavenging activity of supplemented ice creams were greater than control. Peroxide value of 60 days old ice cream was less than allowable limits (10 MeqO_2_/kg). The innovative technologies to protect olein fraction supplemented dairy products using adequate preparation and/or packaging are needed. Industrially, further research should be conducted which would utilize the olein fraction of chia oil for the development of functional foods, or medicinal, pharmaceutical and other non-food industrial applications.
